# Dynamic Deformation Measurement of Specular Surface with Deflectometry and Speckle Digital Image Correlation

**DOI:** 10.3390/s20051278

**Published:** 2020-02-26

**Authors:** Yao Hu, Shaopu Wang, Xuemin Cheng, Chengqiang Xu, Qun Hao

**Affiliations:** 1 Beijing Key Lab. for Precision Optoelectronic Measurement Instrument and Technology, School of Optics and Photonics, Beijing Institute of Technology, Beijing 100081, China; huy08@bit.edu.cn (Y.H.); xcq15210538753@163.com (C.X.); 2 Beijing Institute of Spacecraft Environment Engineering, Beijing 100094, China; wangshpu@163.com; 3 Graduate School at Shenzhen, Tsinghua University, Shenzhen 518055, China; chengxm@sz.tsinghua.edu.cn

**Keywords:** deformation measurement, digital image correlation, deflectometry, digital speckle, specular surface, dynamic measurement

## Abstract

The deformation measurement of a specular surface is of great importance during the quality inspection and installation of optical elements or wafers, especially those with large apertures. We propose a deflectometry method with speckle digital image correlation (DeSDIC) to realize the dynamic and high-accuracy measurement of the deformation on specular surfaces, with a simple system structure and robustness to noises and environmental vibrations. Random speckle pattern displayed on liquid crystal display is reflected by the original surface under test (SUT), and the distorted pattern is recorded by a camera. This originally distorted pattern is taken as the reference image, and the patterns captured afterwards are digitally correlated with the reference image to calculate the gradient change and deformation of the SUT. The theoretical relationships and an experimental one-step calibration scheme are proposed. Both static and dynamic deformations of a deformable mirror were experimentally measured to demonstrate the feasibility and accuracy of DeSDIC, which is comparable to phase-measuring deflectometry and interferometry.

## 1. Introduction

Deformation of the solid surface is the consequence of various dynamic physical events, including the strain from stress or temperature change. For example, when mounted with an improper mechanism or subjected to excessive force, optical elements will suffer from surface deformation or non-uniform in the refractive index, which will greatly affect the performance of the elements. The measurement of such deformation is an effective approach for digging into the corresponding physical process and furthermore guaranteeing fine surface quality, appropriate mounting and environment control.

Deformation is the change in the surface shape; thus, various methods for surface shape or figure error measurement can help solve the problem. Interferometry or deflectometry is among the effective approaches for surface measurement of optical elements. Commercial interferometers from Zygo in the US, XONOX in Germany, and other manufacturers have been popularly used in both laboratory and the industrial field. However, interferometers are generally expensive and complicated, requiring careful environmental control. As a typical kind of delectometry, phase-measuring deflectometry (PMD) [1] with sinusoidal fringes [2,3,4] or binary pattern [5] has been developed for a decade for surface measurement from slopes or gradients. Particularly, software-configurable optical test system [6,7] based on PMD has been proposed for measuring large aspherical surfaces rapidly, robustly, and accurately, similar to other kinds of image-based measurement methods [8]. Accurate phase measurement [9], systematic calibration [10,11], and three-dimensional (3D) reconstruction from slope information [12,13,14] are among the active topics. PMD has also been proposed for deformation measurement [15], and the accuracy requirements on calibration can be lowered. Most PMD methods adopt phase shifting for phase retrieval, which needs more than one image. Thus, dynamic PMD [16,17] has also attracted extensive attention.

If we do not pay attention to the absolute surface shape at each moment but focus only on the deformation, we can adopt techniques in mechanical testing, e.g., digital image correlation (DIC) [18,19,20,21]. Two-dimensional (2D) DIC calculates the in-plane displacement with laser speckles formed on the surface or artificial speckles painted or projected on the surface. The accuracy for 2D DIC relies on image processing and can be as high as nanometer scale when the speckle size is of several microns and sub-pixel algorithm is employed [22]. 3D DIC can measure both in-plane and out-of-plane displacement using stereo-correlation, and the measurement accuracy depends on the field of view. A combination of DIC and fringe projection [23,24] has recently been proposed for the extraction of 3D displacement. The abovementioned methods work well for scattering materials, including industrial materials such as rubber, metal and unpolished glass, and even biological tissues such as bones and vessels. Moreover, because only one frame of an image is necessary, the above methods all have potential use for dynamic measurement [25,26,27]. For an optical surface after a good polish, if no modification is allowed to the surface for protection, minimal scattering occurs and nearly no speckle can be generated on the surface under test (SUT) only with laser illumination. Thus, the DIC method is not available. To solve this issue, PMD-based speckle pattern shifting deflectometry based on DIC [28,29] has been applied to flat specular surface shape measurement because of its robustness over noises compared with PMD with sinusoidal fringes and phase shifting algorithms [30]. Precise systematic calibration is still critical for micron-level accurate shape measurement.

We report a combination of deflectometer setup and speckle DIC algorithm (DeSDIC for short) to realize high-accuracy dynamic deformation measurement of specular surface with a simple and low-cost system. The theoretical relationship between the deformation and speckle displacement is deduced and an experimental one-step calibration method is proposed. The static deformation of a deformable mirror (DM) was measured and the result was compared with that obtained by a commercial interferometer. Finally, dynamic measurements of DM deformations demonstrated the feasibility and accuracy of the proposed method.

## 2. Materials and Methods

**Figure 1:**
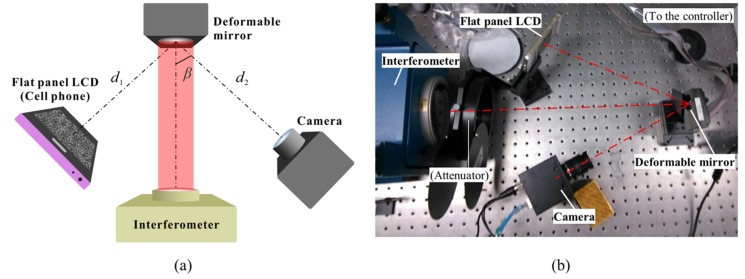
Setup of the DeSDIC demonstration system for measuring the deformation of the deformable mirror (top view). The camera records the reflected speckle pattern displayed on the flat-panel LCD (a cell phone screen in our case). The interferometer is for deformation measurement result comparison. (**a**) The schematic setup. (**b**) The photo of the experimental system.

The setup of the DeSDIC system is shown in Figure 1. A flat-panel liquid crystal display (LCD), which is actually a cell phone screen in our case, with an effective size of 90 × 50 mm and a resolution of 1920 × 1080 pixels displays an image of a high-contrast speckle pattern generated with the algorithm from Ref. [31]. The SUT is a DM (OKO, 15-mm 37-channel MMDM) with a clear aperture of 15 mm driven by 37 channels of piezo actuators arranged in a circular manner. The maximum stroke of a single actuator is 8 μm. The speckle pattern is reflected by the DM and recorded by a monochromatic charge coupled device (CCD) camera (Microview, MVC1450DAM-GE15). The focal lens of the non-telecentric objective lens (Computar, M3514-MP) is *f* = 35 mm and F number is 1.4. The CCD chip has a size of 1/2 inch, a pixel size of 4.65μm, and a resolution of 1392 × 1040 pixels. The field of view is about 40 mm × 30 mm in the DM plane, and the pixel number along the diameter of the DM is around 520. The distance between the cell phone and the DM *d*_1_ and that between the DM and the camera *d*_2_ are about 280 and 320 mm, respectively. The included angle *β* between the normal of the DM and the optical axis of the camera is about 30°. The interferometer in Figure 1 (Zygo Dynafiz 4’’) is for experimental comparison of the measured deformation of the DM.

Unlike PMD, DeSDIC does not measure the phase but uses DIC to calculate the pattern distortion caused by the local slope change of the DM. Thus, the deformation is reconstructed in four steps: (1) quantifying local pattern displacement from two speckle images with DIC; (2) measuring the rotational angle of a facet from the local distortion; (3) calculating the gradient of the deformation from the rotational angle; and (4) reconstructing the deformation from the gradient. The detailed mathematical analysis is proposed as follows.

**Figure 2:**
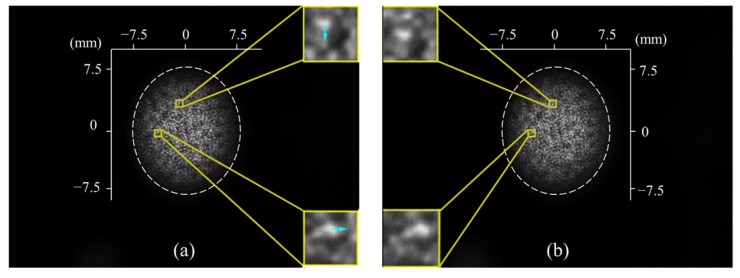
Typical recorded speckle pattern images (**a**) before and (**b**) after the deformation. The yellow boxes mark two pairs of subsets with 25 × 25 pixels in the two images. In the magnified images, obvious shifts along the arrows can be observed. Quantitative displacement can be calculated with DIC. The dashed white ellipses indicate the active regions for the speckle DIC processing.

Step 1: DIC is adopted to calculate the 2D displacement (Δ*u*, Δ*v*) of the speckle. In detail, we denote the intensity distribution of the recorded speckle pattern image before and after the deformation to be *S*_0_(*u*, *v*) and *S*_1_(*u*, *v*). Figure 2 shows typical *S*_0_(*u*, *v*) and *S*_1_(*u*, *v*) in the full field of view of the camera. Little information can be retrieved by the naked eye. Thus, from the two images, we sample two square subsets denoted as *g*(*u*, *v*) and *h*(*u*, *v*) centered at (*u*_0_, *v*_0_) with a size of (2*m* + 1) × (2*m* + 1) pixels, where *m* is an integer. In Figure 2, the subsets are marked with yellow boxes and magnified, and *m* = 12. Obvious shifts along the arrows can be noticed. For the quantitative displacement, we adopt an established DIC method: the zero-normalized cross-correlation of the two subsets [19]

(1) $$C_{\mathrm{ZNCC}}=\sum_{i=-m}^{m}\sum_{j=-m}^{m}\left\{\frac{\left[g(u_{i},v_{j})-g_{\mathrm{mean}}\right]\times \left[h(u_{i},v_{j})-h_{\mathrm{mean}}\right]}{\Delta g\Delta h}\right\},$$

where $g_{\mathrm{mean}}=\frac{1}{{\left(2m+1\right)}^{2}}\sum_{i=-m}^{m}\sum_{j=-m}^{m}g(u_{i},v_{i})$, $h_{\mathrm{mean}}=\frac{1}{{\left(2m+1\right)}^{2}}\sum_{i=-m}^{m}\sum_{j=-m}^{m}h(u_{i},v_{i})$, $\Delta g=\sqrt{\sum_{i=-m}^{m}\sum_{j=-m}^{m}{\left[g(u_{i},v_{i})-g_{\mathrm{mean}}\right]}^{2}}$, and $\Delta h=\sqrt{\sum_{i=-m}^{m}\sum_{j=-m}^{m}{\left[h(u_{i},v_{i})-h_{\mathrm{mean}}\right]}^{2}}$.

A sub-pixel interpolation scheme can be utilized before the similarity between the two subsets is evaluated [19]. Finally, the first-order displacement (Δ*u*, Δ*v*) of the speckle pattern between the two subsets can be obtained from the correlation peak shift. The center of the subset scans over the whole image and the displacement distribution over the whole aperture can be obtained. The immediate result from DIC is in pixel unit and can be converted to linear displacement with the pixel size of the detector. The size of the subsets should be compromised between the accuracy and the computation time.

**Figure 3:**
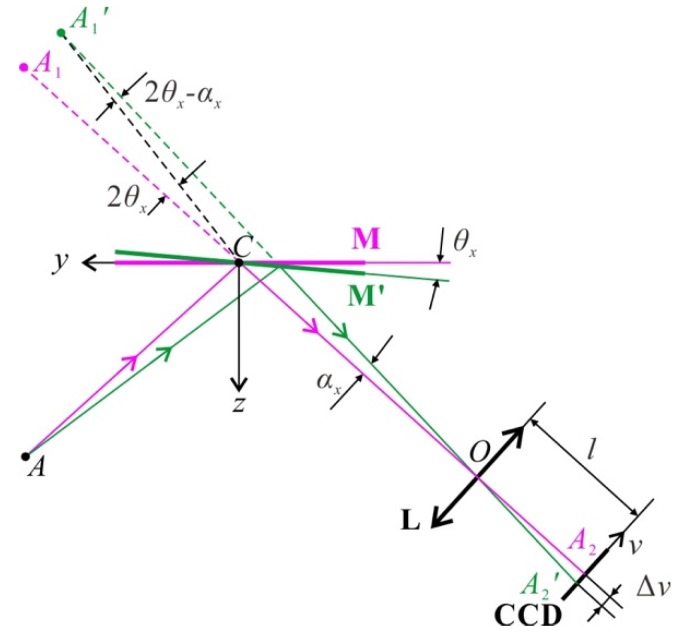
Imaging geometry for measuring the rotation angle *θ_x_* of mirror element **M** from the image displacement Δ*v*. *A* is the object point. **M** rotates around its center *C* by an angle *θ_x_* to **M’**. *A*_1_ and *A*’_1_ are the virtual images of *A* by mirror **M** and **M’**, respectively. *L* is the objective lens and *O* is its optical center. *A*_2_ and *A*’_2_ are the images of *A*_1_ and *A*’_1_ on the CCD sensor. The displacement between *A*_2_ and *A*’_2_ is Δ*v*.

Step 2: The rotation angle (*θ_x_*, *θ_y_*) of a facet **M** is measured from the image displacement. Figure 3 shows the basic geometry. The object point denoted as *A*, e.g., a point in the speckle displayed by the cell phone, is virtually imaged to *A*_1_ by the mirror **M**. Then, *A*_1_ is imaged to *A*_2_ on the CCD sensor by the objective lens *L*. *O* is the optical center of *L*. We set a Cartesian coordinate system at the center of the mirror to describe its rotation, with the normal as *z*-axis and the direction perpendicular to the plane *ACO* as *x*-axis. An image coordinate system *uv* is set at the CCD image plane with *u*-axis parallel to *x*-axis.

If **M** is rotated around the *x*-axis by an angle *θ_x_* to **M’**, then the virtual image *A*_1_ will be rotated around *C* by angle 2*θ_x_* to *A*’_1_, i.e., ∠*A*_1_*CA*’_1_=2*θ_x_*. Then *A*’_1_ is imaged to *A*’_2_. The angle included between the imaging rays for *A*_2_ and *A*’_2_ is denoted as *α_x_*. According to simple geometry, in triangle *A*’_1_*CO*, we have

(2) $$|A_{1}^{\prime }C|/\sin\alpha_{x}=|CO|/\sin\left(2\theta_{x}-\alpha_{x}\right),$$

where|*A*’_1_*C*| = |*AC*| = *d*_1_, and |*CO*| = *d*_2_. If *θ_x_* is small enough (less than 1° degree, for example), then we have

(3) $$\alpha_{x}=2d_{1}\theta_{x}/\left(d_{1}+d_{2}\right),$$

Given the focal length of *L* as *f*, the image distance *l* between *O* and the CCD plane can be calculated from the object distance (*d*_1_ + *d*_2_) as

(4) $$l=f\left(d_{1}+d_{2}\right)/\left(d_{1}+d_{2}-f\right),$$

Then, the image shift Δ*v* due to the rotation *θ_x_* equals

(5) $$\Delta v=l\tan\alpha_{x},$$

and substituting Equations (3) and (4) into Equation (5) yields

(6) $$\theta_{x}=k\left(d_{1}+d_{2}-f\right)\Delta v/2d_{1}f,$$

where *k* is a calibrated coefficient because the distance and focal length are estimated. If **M** is rotated around *y*-axis, then *θ_y_* will have a similar relationship with the image shift Δ*u*. The detailed calibration method is shown in the experimental result section. Moreover, because of the off-axial configuration, the circular aperture is distorted into a standing ellipse in Figure 2 and should be restored according to the angle *β* in Figure 1.

Step 3: The gradient change $\left(\Delta W_{x}^{\prime },\Delta W_{y}^{\prime }\right)$ is calculated from the rotational angle (*θ_x_*, *θ_y_*). If we consider **M** a facet on the DM, then its rotation is caused by the deformation of the mirror, and then the angle *θ_x_* or *θ_y_* will be equal to the local normal direction change in *x* or *y*-direction. With the surface shape of the measured DM before and after the deformation denoted as *W*_1_(*x*, *y*) and *W*_2_(*x*, *y*), respectively, then Δ*W*(*x*, *y*) = *W*_1_ − *W*_2_ is the deformation we want to measure, where (*x*, *y*) is omitted for concision. Considering the physical property of a deformation mirror, *W*_1_ and *W*_2_ are continuous and differentiable. The normal vector of *W*_1_ and *W*_2_ can be formulated as

(7) $$\left\{ \begin{array}{l} N_{1}=\left(W_{1x}^{\prime },W_{1y}^{\prime },1\right)\\ N_{2}=\left(W_{2x}^{\prime },W_{2y}^{\prime },1\right) \end{array}\right..$$

The normal vector of Δ*W* is

(8) $$T=\left(\Delta W_{x}^{\prime },\Delta W_{y}^{\prime },1\right)=\left(W_{2x}^{\prime }-W_{1x}^{\prime },W_{2y}^{\prime }-W_{1y}^{\prime },1\right).$$

According to basic geometry, the angle *θ_x_*, which is equal to the angle between *N*_1_ and *N*_2_ in the *x*-direction, is related to the components of the vectors as

(9) $$\tan\theta_{x}=\left(W_{1y}^{\prime }{}^{-1}-W_{2y}^{\prime }{}^{-1}\right)/\left(1+W_{1y}^{\prime }{}^{-1}W_{2y}^{\prime }{}^{-1}\right).$$

If the rotation angle is small enough, we have $\tan\theta_{x}\approx \theta_{x}$ and $W_{1y}^{\prime }\approx W_{2y}^{\prime }$. Then, Equation (9) can be rewritten as

(10) $$\theta_{x}=\Delta W_{y}^{\prime }/\left(1+W_{1y}^{\prime }{}^{2}\right).$$

Together with Equation (6) we have

(11) $$\Delta W_{y}^{\prime }=\left(1+W_{1y}^{\prime }{}^{2}\right)k\frac{d_{1}+d_{2}-f}{2d_{1}f}\Delta v.$$

Similarly,

(12) $$\Delta W_{x}^{\prime }=\left(1+W_{1x}^{\prime }{}^{2}\right)k\frac{d_{1}+d_{2}-f}{2d_{1}f}\Delta u.$$

Foreknowledge of the surface shape *W*_1_ is required here, which can be obtained from its nominal shape or rough measurement in advance. 

Step 4: The deformation Δ*W* is constructed from the partial derivatives with Tikhonov deconvolution in Ref. [14,32] for improved noise immunity as

(13) $$\Delta W=F^{-1}\left\{\frac{i2\pi \left(F\left\{\Delta W_{x}^{\prime }\right\}\lambda +F\left\{\Delta W_{y}^{\prime }\right\}\mu \right)}{-4\pi^{2}\left(\lambda^{2}+\mu^{2}\right)+\gamma }\right\},$$

where *F*{∙} and *F*^−1^{∙} are the Fourier and inverse Fourier transform, respectively, *λ* and *μ* are frequency coordinates, and *γ* is the regularization number.

## 3. Experiments and Results

We conducted three experiments to verify the system and method. First, the rotation angle of the DM was measured to calibrate the system parameter, especially *k*, and verify the angular measurement linearity and accuracy. Second, a static deformation was measured and quantitatively compared with the result from the interferometer to demonstrate the accuracy. Finally, dynamic measurement of the deformation was performed to show the potential in dynamic inspection.

### 3.1. Calibration of the Linear Coefficient

Deformation measurement focuses more on the local slope change rather than the absolute slope; thus, we deduce the approximately linear relationship between slope change (rotation) and the speckle pattern shifting in Equation (6). Consequently, we propose a one-step calibration method for the correction coefficient *k* in Equation (6) because the distance and focal length are estimated. Completely calibrating the internal and external parameters of the imaging system is not necessary. We believe this simple calibration method can contribute to simple structure and fast measurement of small deformation on specular surface.

In the first experiment, the DM in Figure 1 was mounted on a precision rotation platform (BOCIC, MRS201) with an encoder that has an angular measurement resolution of 1” and repeatability of 2”. The rotation angle of the DM and the shift of the speckle were recorded for the calibration of *k*. Figure 2a is the speckle image taken at the initial position. The dashed white ellipses in Figure 2 indicate the active regions for the speckle DIC processing. The boundary of the region was calculated by ellipse fitting of the edge after binarization with 20 as the grayscale threshold. Here the grayscale threshold 20 was manually determined from the sectional intensity distribution in which below 20 the contrast of the speckle could hardly be seen. The speckle image was then stretched in horizontal direction according to the ratio between the lengths of the major and minor axes of the ellipse to restore to a circular aperture. 

**Figure 4:**
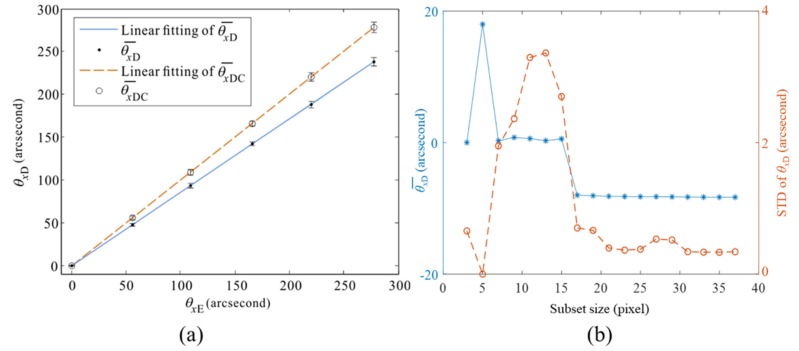
Rotational angle measurement results. (**a**) The results *θ_x_*_D_ measured with DeSDIC are plotted against *θ_x_*_E_ measured with the encoder. The subset size in DeSDIC was optimized to 21 × 21 pixels. ${\bar{\theta }}_{xD}$ is the average of *θ_x_*_D_ and the error bar shows the standard deviation of *θ_x_*_D_ at all the valid pixels inside the DM clear aperture. ${\bar{\theta }}_{x\mathrm{DC}}$ is ${\bar{\theta }}_{xD}$ after correction of the systematic error. The linearity between ${\bar{\theta }}_{xD}$ and *θ_x_*_E_ is nearly 1 and the slope before and after the correction is 0.86 and 1.00, respectively. (**b**) The optimization of subset size in DeSDIC.

The rotational angle measured with the encoder is denoted as *θ_x_*_E_, which is treated as the true value and plotted as the horizontal axis in Figure 4a. Also the angles at all the pixels inside the DM clear aperture measured with DeSDIC setup and Equation (6), where *k* is preset to be 1, are denoted as *θ_x_*_D_(*u*, *v*), the average of which is denoted as ${\bar{\theta }}_{xD}$. 

The calibration results are plotted in Figure 4a, where the solid dots show ${\bar{\theta }}_{xD}$ and the error bars show the standard deviation (STD) of 131172 valid points. When *θ_x_*_E_ increases from 56” to 278”, the average Δ*v* changes from 1.62 pixels to 8.46 pixels, and STD varies from 2.05” to 4.90”, corresponding to 0.07 pixels and 0.18 pixels. The accuracy of DIC decreases with the increment of the rotational angle because of the decorrelation of the speckles, which has been an important problem in DIC and will be focused in our future work. 

The blue solid line in Figure 4a is the linear fitting of ${\bar{\theta }}_{xD}$ with a slope of 0.86 and R^2^ of nearly 1. This finding indicates that a systematic error exists, and we calibrate the linear coefficient in Equation (6) as *k* = 1/0.86. The corrected ${\bar{\theta }}_{xD}$ is denoted as ${\bar{\theta }}_{x\mathrm{DC}}$ and plotted as hollow circles in Figure 4a. The orange dashed line is the linear fitting of ${\bar{\theta }}_{x\mathrm{DC}}$ and the norm of the residuals is 3.07” (14.9 μrad), which can be regarded as the calibration error. The maximum STD of $\theta_{x\mathrm{DC}}$ increases to 5.75” (27.9 μrad), which can be regarded as the angular measurement accuracy of the current DeSDIC system. The experimental accuracy is close to that of the phase-shifting PMD [1].

The subset size in DIC was optimized for low measurement error and yet relatively short computational time. The average value and STD of *θ_x_*_D_ when *θ_x_*_E_ = 278” is plotted against the subset size in Figure 4b. The average becomes stable when the subset size is greater than 17 pixels. The STD first increases and then reduces with the increase in the subset size and became stable when the subset size is greater than 21 pixels. Thus, the subset size was optimized to be 21 × 21 pixels.

### 3.2. Deformation Measurement

**Figure 5:**
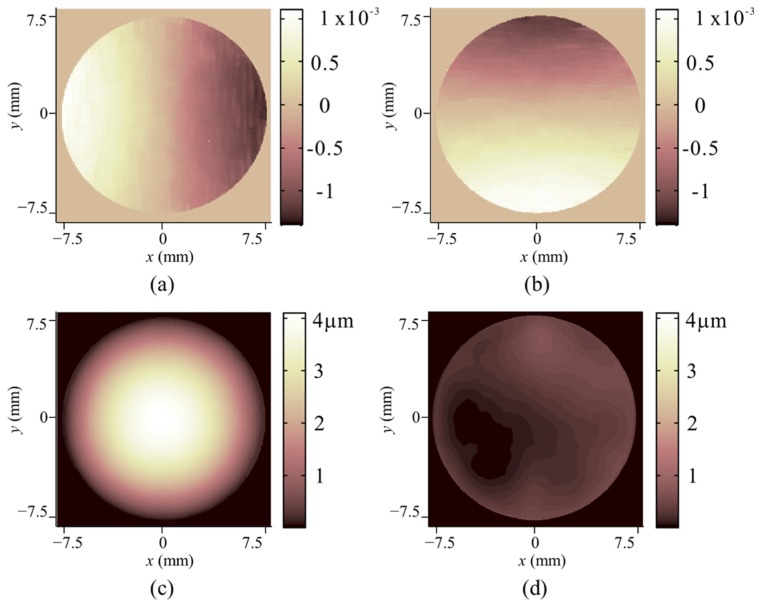
Deformation measured with interferometer and the proposed system. (**a**) gradient change in the horizontal direction measured with DeSDIC; (**b**) gradient change in the vertical direction measured with DeSDIC; (**c**) deformation measured with DeSDIC; and (**d**) point-to-point difference between the deformation maps measured with the two methods. The color bar of (**c**) and (**d**) is set the same to intuitively show the magnitude of the error.

In the second experiment, the DM was controlled to change the surface shape. The deformation was simultaneously measured by an interferometer and the proposed system. The surface shape of the DM before the deformation *W*_1_ was measured with the interferometer, and it was a bowl shape with a peak-to-valley (PV) value of 8.34 λ (λ = 632.8 nm) corresponding to 5.28 μm. Thus, $W_{1y}^{\prime }{}^{2},W_{1x}^{\prime }{}^{2}$ in Equations (11) and (12) can be neglected. Generally, for most DMs with planar original shapes, the original slopes can be treated as 0 as well. However, if the SUT exhibits an obvious undulation, then the original shape should be known beforehand and a telecentric camera lens should be adopted to avoid defocusing. The speckle images before and after the deformation are shown in Figure 2, and the measurement results are shown in Figure 5. The gradient change in the horizontal and vertical directions measured with DeSDIC are shown in Figure 5a,b, respectively. The maximum local slope angle change was 1.2 mrad or 247”. The PV value of the deformation measured with DeSDIC in Figure 5c was 4.04 μm, while that measured with a Zygo interferometer was 3.93 μm. The difference was about 0.1 μm, and the shape of the deformation was similar to each other. The point-to-point difference between the deformation maps measured with the two methods is displayed in Figure 5d, the PV and root-mean-square (RMS) value of which is 0.7 μm and 0.1 μm, respectively. This finding can be regarded as the deformation measurement accuracy of the DeSDIC system.

### 3.3. Dynamic Measurement

**Figure 6:**
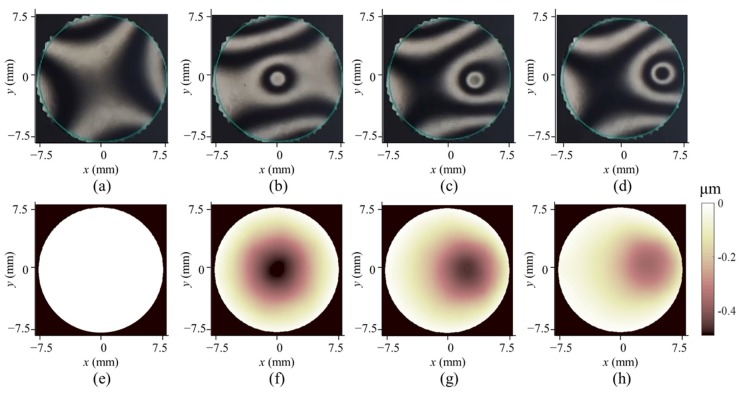
Four frames of typical interferograms and the corresponding dynamic deformations measured with DeSDIC. (**a**–**d**) are the interferograms and (**e**–**h**) are the measured deformations. (**a**) is the interferogram corresponding to the original DM shape.

Finally, the DM was controlled as follows to change the surface shape at 5 Hz. The 37 actuators were set to the maximum voltage one by one with all the other actuators set to 0. The changing time was shorter than 1 ms; thus, the surface was almost static between two changes. A video of the deformed speckles was recorded at a frame rate of 15 fps, and the images were extracted from the video for deformation reconstruction. As long as the exposure time is short enough to record clear speckle images without any motional blur, the dynamic deformation, i.e., the instantaneous off-plane deformation of the surface related to the original shape, can be correctly measured with DeSDIC. The deformation is dynamic and cannot be continuously measured with the phase-shifting interferometer; thus, we recorded only the corresponding interferograms for comparison. Four frames of typical interferograms and measured deformation results with DeSDIC are shown in Figure 6. Among them, Figure 6a,e is corresponding to the original surface. The positions of the actuators are in good correspondence. The PV values of the deformation in Figure 6f–h are 0.484, 0.445, and 0.356 μm, respectively. Supplementary Video S1 is a better demonstration of measuring dynamic deformation with DeSDIC.

### 3.4. Error Analysis

Considering the error analysis of the system and method, we have to mention three main error sources. 

First, the theoretical model in Equations (3) and (11) introduced the principle error. The small angle approximation limited the local slope angle change measurement range to less than 1° or 17.5 mrad, with a maximum principle relative error of 1 × 10^−4^. If the local slope angle change increases to 5°, the maximum principle relative error will grow up to 2 × 10^−3^. Deflectometry measurement relies on the accuracy of slope measurement and DeSDIC inherits this feature. Second, the size of the subset in DIC guaranteed the accuracy, as shown in Figure 4b, but reduced the spatial resolution. If the local speckle displacement is unknown, then a larger subset size is preferred to cover the speckle displacement and avoid calculation error due to complete decorrelation. However, if the local speckle displacement (slope change) is small and varying fast from pixel to pixel, then a large subset will average the slope change and smooth deformations of high spatial frequency. Third, the aberration of the imaging lens will introduce measurement errors. When the deformation increases, defocus will occur, and the contrast of the speckle will decrease. Also, the distortion or field curvature will change the distribution or contrast of the speckle at the edge of the field of view.

According to the above analysis, possible improvements are as follows: When applied in a large deformation situation, a more accurate model should be derived, and a telecentric lens should be adopted. Distortion should be corrected with a better lens or a software algorithm. Co-axis configuration, smaller speckle size [33], and better DIC algorithm can improve the accuracy. We will focus on these problems in future work.

## 4. Conclusions

We proposed a combination of deflectometer setup and speckle DIC algorithm to realize high-accuracy dynamic deformation measurement of specular surface with a simple and low-cost system. A linear model of the local slope change and the speckle displacement is deduced. A one-step calibration scheme based on the model is proposed for measuring the deformation with a maximum local slope change less than 1°, which makes completely calibrating the internal and external parameters of the imaging system not necessary. The current system realized a slope measurement precision of 5.75” (27.9 μrad) and deformation RMS measurement accuracy of 0.1 μm, which is comparable to traditional PMD and interferometry. Continuous deformation of the DM was also measured to demonstrate its potential in dynamic measurement. This method and system are applicable in the measurement of fast and small deformation on specular surfaces with a very simple system structure and calibration procedure.

## 5. Patents

The authors have been authorized a Chinese patent ZL201510810141.4 resulting from the work reported in this manuscript.

## Supplementary Materials

The following is available online at https://www.mdpi.com/1424-8220/20/5/1278/s1, Video S1: Dynamic deformations of a deformable mirror measured with DeSDIC: comparative interferogram and corresponding deformation.
